# Increasing entropy for colloidal stabilization

**DOI:** 10.1038/srep36836

**Published:** 2016-11-22

**Authors:** Songping Mo, Xuefeng Shao, Ying Chen, Zhengdong Cheng

**Affiliations:** 1 Guangdong Provincial Key Laboratory on Functional Soft Condensed Matter, School of Materials and Energy, Guangdong University of Technology, Guangzhou 510006, China; 2 Artie McFerrin Department of Chemical Engineering, Texas A &M University, College Station, Texas 77843-3122, USA

## Abstract

Stability is of paramount importance in colloidal applications. Attraction between colloidal particles is believed to lead to particle aggregation and phase separation; hence, stability improvement can be achieved through either increasing repulsion or reducing attraction by modifying the fluid medium or by using additives. Two traditional mechanisms for colloidal stability are electrostatic stabilization and steric stabilization. However, stability improvement by mixing attractive and unstable particles has rarely been considered. Here, we emphasize the function of mixing entropy in colloidal stabilization. Dispersion stability improvement is demonstrated by mixing suspensions of attractive nanosized titania spheres and platelets. A three-dimensional phase diagram is proposed to illustrate the collaborative effects of particle mixing and particle attraction on colloidal stability. This discovery provides a novel method for enhancing colloidal stability and opens a novel opportunity for engineering applications.

The concern for dispersion stability is significant in colloidal dispersions. A colloidal dispersion consists of colloidal particles dispersed in a continuous fluid medium. Colloidal dispersions possess considerable application potential in various fields, including food, soil science, hydrology, catalysis, agrochemical, pharmaceutical, cosmetics, environmental science and technology, composite materials manufacturing and energy industries^1^,^2^,^3^,^4^,^5^,^6^,^7^,^8^.

In general, the attraction between colloidal particles leads to particle aggregation and phase separation of colloidal dispersions. Stability improvement can be achieved through either increasing repulsion or decreasing attraction between colloidal particles. Two traditional mechanisms for colloidal stability are electrostatic stabilization and steric stabilization. Electrostatic stabilization is the mechanism in which the attractive van der Waals forces are counterbalanced by the repulsive Coulomb forces acting between the charged colloidal particles. The steric stabilization of colloids involves additives that are added to the fluid medium to inhibit the coagulation of the particle suspension. The traditional methods for stability improvement include changing the property (such as pH value^9^) of the fluid medium or using additives. Additives usually include dispersants, such as surfactants and polymer^9^,^10^,^11^. Charged particles^12^,^13^,^14^,^15^,^16^ can also act as dispersants for colloidal stabilization in multi-component suspensions. Dispersants should be stably dispersed in the fluid medium to achieve good dispersion stability of colloidal particles. However, can the effect of mixing itself possibly contribute to the improvement of colloidal stability regardless of the stability of the dispersants?

For a colloidal dispersion of multi-component particles, the free energy change by particle mixing can be used to study colloidal stability. A system becomes more stable at lower free energy, *G*, which is defined as follows^17^:


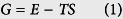


where *T*, *E* and *S* are the temperature, energy and entropy of the system, respectively. According to Equation (1), free energy decreases with decreasing energy or increasing entropy. The free energy change after mixing multi-component particles, Δ*G*_mix_, can be calculated as follows:


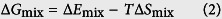


where Δ*E*_mix_ = *E*_mix_ − *E*_demix_, Δ*S*_mix_ = *S*_mix_ − *S*_demix_ are the energy and entropy change of the system after mixing multi-component particles, respectively, the subscript mix and demix denotes the state after mixing and demixing (or before mixing), respectively. Since a negative change in free energy is thermodynamically favorable, if Δ*G*_mix_ < 0, which means that mixing will decrease the free energy of the colloidal dispersion, mixing will be a spontaneous process and results in good dispersion of the multi-component particles. Otherwise if Δ*G*_mix_ > 0, demixing is thermodynamically favored to decrease the free energy of the colloidal dispersion.

Although the entropically driven phase behaviours of binary colloidal dispersion systems have garnered considerable attention^18^,^19^,^20^,^21^,^22^,^23^,^24^,^25^,^26^, these works have focused on phase demixing^26^,^27^, and the function of entropy in the improvement of colloidal stability has been scarcely reported until now.

To evaluate the function of mixing entropy in colloidal stability, we mixed two types of particles and studied the phase behaviour of the binary suspension. To minimize the effects of electrostatic stabilization and steric stabilization, we mixed two types of unstably dispersed particles with electrostatic attractions. Moreover, to minimize other stabilization effects related to the difference in chemical composition, if any, we selected TiO_2_ spheres and platelets, which are particles with the same chemical composition but different shapes, as model particles for mixing.

## Results and Discussion

**Figure 1:**
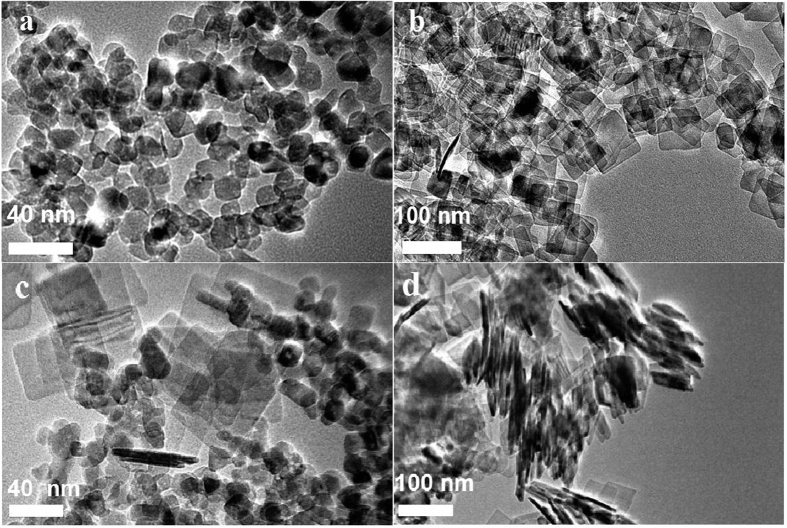
Transmission electron microscopy (TEM) photographs of TiO_2_ particles. (**a**) Spheres. (**b**) Platelets. (**c**) Particle mixture in the upper phase of the demixing binary suspension. (**d**) Particle mixture in the sedimentation phase of the demixing binary suspension.

The average diameter of TiO_2_ spheres is approximately 20 ± 10 nm, and the TiO_2_ platelets are generally rectangular with the edge length in the range of 20–80 nm, as shown in Fig. 1a,b. The spheres and platelets were dispersed into deionized water having a pH value of 7.0. The zeta potentials of the sphere and platelet suspensions, which were measured by a zeta potential analyzer, were 5.73 and −4.11 mV, respectively. This finding indicates that the suspensions were unstably dispersed because of low absolute zeta potentials^28^; moreover, the electrical charges on spheres and platelets are positive and negative, respectively, thus the electrostatic forces between the spheres and platelets will be attractive if the spheres and the platelets are mixed.

**Figure 2:**
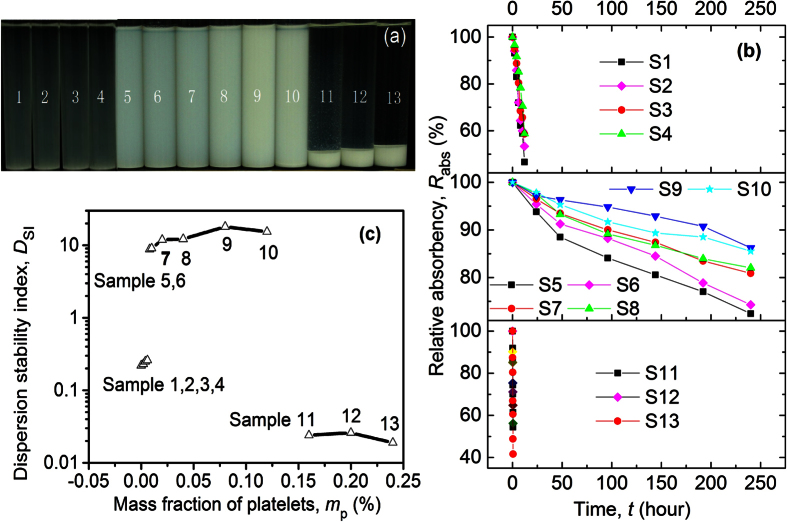
Appearance, absorbency and stability evaluation of binary suspensions at 24 h after preparation. (**a**) Photographs of samples from No. 1 to No. 13; the weight fraction of spheres is 0.1% ± 0.0005%, and the weight fractions of platelets are 0%, 0.002%, 0.004%, 0.006%, 0.008%, 0.01%, 0.02%, 0.04%, 0.08%, 0.12%, 0.16%, 0.20% and 0.24%, with the uncertainty of each sample of ±0.0005%. (**b**) Relative absorbency and (**c**) Dispersion stability evaluation of the samples. The initial ‘S’ in (**b**) denotes sample.

A series of suspensions was prepared with the mass concentrations of spheres ranging from 0.002% to 0.50%, and platelets ranged from 0.002% to 0.24%. The binary suspensions were immobilized for observing the phase transition phenomenon after preparation. Figure 2 shows photographs of suspensions of 0.1 ± 0.001% spheres with platelets ranging from 0.002% to 0.24%. All samples were found to be fluid immediately after preparation. Two phase behaviours were observed at a minimum of 24 h after sample preparation. As shown in Fig. 2a, demixing with an interface observed in sample Nos 1–4 and Nos 11–13, whereas sample Nos 5–10 were stable and were well dispersed.

The optical absorbencies of the suspensions were measured by ultraviolet–visible spectroscopy to quantify dispersion stability. The absorbency variation of each sample was measured. Relative absorbency *R*_abs_ is defined as the ratio of absorbency *A* to the initial absorbency *A*_0_ at the middle of sample container after preparation:


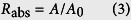


Relative absorbencies of the samples were plotted in Fig. 2b. The dispersion stability of each sample was evaluated by the reciprocal of absolute value of slope of the curve in Fig. 2b, which is defined as the dispersion stability index


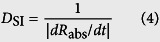


The dispersion stability index of each sample is shown in Fig. 2c as a function of mass fraction of platelets. There are three zones, zone 1 for samples 1 to 4, zone 2 for samples 5 to 10, and zone 3 for samples 11 to 13. The results indicated that the addition of TiO_2_ platelets within the range of 0.008–0.12% improve the dispersion stability of 0.1% TiO_2_ sphere suspension (samples 5–10, zone 2), whereas an excessively small (samples 1–4, zone 1) or large number of platelets (samples 11–13, zone 3) do not induce improvement. In zone 1, samples 1 to 4 have similar dispersion stability because the amount of platelets addition is too small to have sufficient effect on the sphere suspensions. In zone 3, addition of platelets at relatively high concentration even decreased the dispersion stability of sphere suspensions due to depletion attraction between the nanoparticles, thus zone 3 is even lower than zone 1.

**Figure 3:**
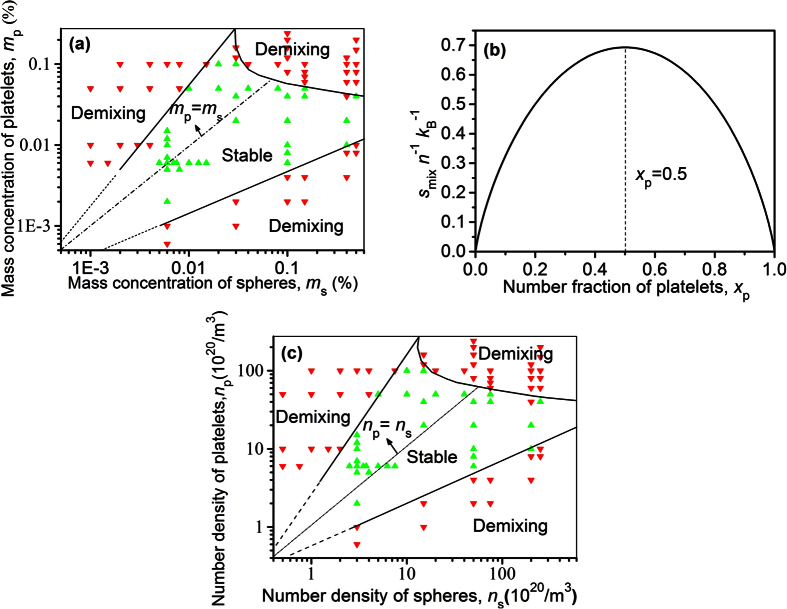
Phase diagram illustrating the stable/demixing regions of the binary suspensions. (**a**) Phase diagram of the mixed platelet and sphere suspensions as a function of mass concentration. (**b**) Dimensionless mixing entropy as a function of number fraction of platelets. (**c**) Phase diagram of mixed platelet and sphere suspensions as a function of number density. Stable phase (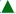) and demixing phase (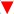) are presented in the phase diagrams.

Two-phase behaviours were also observed at other concentrations of spheres ranging from 0.002% to 0.50%, and platelets ranging from 0.002% to 0.24%. A dynamic phase diagram (Fig. 3a) was constructed based on experimental observations. The salient feature of the phase diagram is the stable mixing region, in which spheres and platelets coexist stably. The stable/demixing division lines at concentrations of spheres and platelets that are lower than 0.002% are presented by dotted lines because of the lack of data given that experimental uncertainty would increase at such or lower concentrations. The results show that whether the binary suspension of spheres and platelets is stable depends on the mass concentrations of the spheres and platelets (*m*_s_ and *m*_p_, respectively). The mixing of platelets with spheres improves the dispersion stability at proper concentrations.

At relatively high *m*_s_ and *m*_p_, the binary suspensions were unstable (upper right region in Fig. 3a), and demixing occurred, which can be attributed to depletion attraction. Similar to the demixing mechanism for a colloidal rod–plate mixture^26^, the origin of demixing may be the excess excluded volume of platelet–sphere pair compared with platelet–platelet and sphere–sphere pairs. The TEM photographs of the supernatant and sedimentation of sample 12 are shown in Fig. 1c,d, respectively. The supernatant consists of both platelets and spheres (Fig. 1c), whereas sedimentation mainly consists of platelets (Fig. 1d). These findings confirm that the attractive depletion force induced by spheres result in the aggregation and sedimentation of platelets.

At relatively low *m*_s_ and *m*_p_, the stability of the suspensions varies with the weight fraction, *f*_p_ = *m*_p_/(*m*_p_ + *m*_s_), of platelets in the sphere–platelet mixture. As shown in Fig. 3a, stable suspensions were observed at a medium *f*_p_ in contrast with the demixing region, in which the particles tend to settle down at either relatively low *f*_p_ or high *f*_p_.

The influence of mixing fraction of spheres and platelets on the dispersion stability of the suspensions is discussed as follows. According to Equation (2), free energy change decreases with decreasing energy change or increasing entropy change. For mixtures of spheres and platelets, the energy of mixing, Δ*E*_mix_, can be obtained similarly to the literature^29^ as follows:


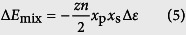


where *z* is the coordination number; *n* = *n*_p_ + *n*_s_ is the total number density of particles; and *x*_p_ = *n*_p_/*n* and *x*_s_ = *n*_s_/*n* are the number fractions of platelets and spheres, respectively; the number density of the platelets and spheres are *n*_p_ = *m*_p_*ρ*_cs_/(*ρv*_p_) and *n*_s_ = *m*_s_*ρ*_cs_/(*ρv*_s_), respectively, where *v*_p_ and *v*_s_ denotes the average volume of a platelet and a sphere, respectively, *ρ* and *ρ*_cs_ is the density of the particles and colloidal suspension, respectively; Δ*ε* is the interaction energy between particles:


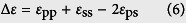


where *ε*_pp_, *ε*_ss_ and *ε*_ps_ refer to the interaction energy between the platelet–platelet, sphere–sphere and platelet–sphere pairs, respectively. The entropy change after mixing, Δ*S*, can be obtained by extending the Onsager theory similarly to the literature^30^,^31^ as follows:


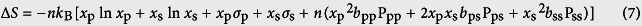


where *k*_B_ is the Boltzmann constant; *σ*_p_ and *σ*_s_ reflect the orientational entropies, which are functions of particle orientational distribution of the platelets and spheres, respectively. The variables *b*_pp_*P*_pp_, *b*_ps_*P*_ps_ and *b*_ss_*P*_ss_ represent the orientational distribution function dependent on excluded volumes of two platelets, a platelet and a sphere and two spheres, respectively. The entropy change in Equation (7) consists of three terms related to mixing, orientation and excluded volumes.

The interaction energy between particles as shown in Equation (6) is difficult to determine; meanwhile, the balance among the three entropy terms in Equation (7) is subtle, and calculation in theory or by simulation presents a formidable challenge^30^. However, the mixing entropy Δ*S*_mix_, that is, the change in entropy related to mixing, can be simply obtained^17^,^30^,^31^ as follows:


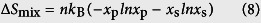


The dimensionless mixing entropy, 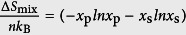, is plotted according to Equation (8) and shown in Fig. 3b. The result indicates that mixing particles increases the entropy of the suspension. The dimensionless mixing entropy first increases and then decreases as *x*_p_ increases, reaching the maximum at *x*_p_ = 0.5. According to Equation (1), free energy decreases with increased entropy. Given that a system becomes more stable at lower free energy, the mixing entropy favours the improvement in dispersion stability of colloidal suspensions.

Considering that the dimensionless mixing entropy reaches its maximum at *x*_p_ = 0.5 for given number density of particles *n*, we plotted a phase diagram versus the number density of the platelets and spheres, as shown in Fig. 3c, with the line *x*_p_ = 0.5 present. For comparison, the line *f*_p_ = 0.5 (corresponding to *m*_p_ = *m*_s_) is also shown in Fig. 3a. Interestingly, the line *f*_p_ = 0.5 does not divide the stable region into two equal parts, with smaller stable region above the line compared with the larger region below the line. By contrast, the line *x*_p_ = 0.5 divides the stable region into two parts of similar size. The coincidence implies that mixing entropy may perform a dominant function in the dispersion stability of binary suspensions. At relatively low *x*_p_ or high *x*_p_ (corresponding to relatively low *f*_p_ or high *f*_p_), the mixing entropy is low (Fig. 3b) and tend to result in positive free energy change for given temperature and energy change according to Equation (2). Since positive free energy change due to mixing is unfavourable for stable dispersion of a colloidal system, the spheres and platelets tend to separate, then aggregate and settle down, in accordance with the phase diagram as shown in Fig. 3a,c.

With increasing concentration, the effect of interaction energy on the stability of colloidal suspension is less effective compared to mixing entropy, that’s why the stable/demixing division lines do not parallel the *x*_p_ = 0.5 or *f*_p_ = 0.5 line (Fig. 3a,c). Interaction energy of particles before and after mixing both increase with increased concentration, but mixing would not change the energy density because the temperature of the suspension is unchanged for the following reason. The concentration of particles in the colloidal suspensions is normally very low. There are many molecules from the continuous phase (in our case is water). So the temperature is determined by the continuous phase which was maintained at constant room temperature. In contrast, mixing increases entropy, and higher mixing entropy Δ*S*_mix_ can be obtained by increasing either number density of particles (*n*) or (−*x*_p_*lnx*_p_ − *x*_s_*lnx*_s_) according to Equation (8). Thus at higher *n*, a lower (−*x*_p_*lnx*_p_ − *x*_s_*lnx*_s_) is enough to obtain the required mixing entropy Δ*S*_mix_ for a negative free energy change (corresponding to stable mixed suspension) according to Equation (2). The (−*x*_p_*lnx*_p_ − *x*_s_*lnx*_s_), which is the dimensionless mixing entropy (Δ*S*_mix_ *n*^−1^ *k*_B_^−1^) according to Equation (8), is lower at relatively higher or lower number fraction of platelets (or spheres) as shown in Fig. 3b. Therefore, with increased nanoparticle concentration (corresponding to increased *n*), the suspensions can be stable even at relatively higher or lower number fraction of platelets (or spheres), resulting in wider stable region between the stable/demixing division lines (Fig. 3a,c).

To quantitatively understand the boundaries between stable suspension and demixing regions as shown in Fig. 3, one would have to calculate also the particle interaction term Δ*E*_mix_ in Equation (2) or Equation (5), which is indeed difficult as we mentioned above. It would be the challenge of future theoretical study.

**Figure 4:**
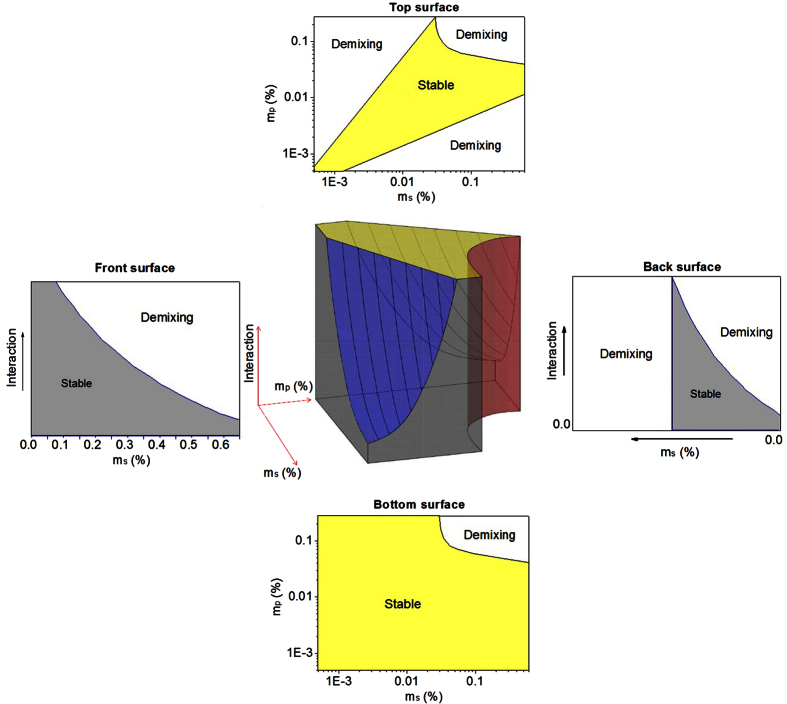
3D phase diagram and its 2D surfaces illustrating the stable and demixing regions of the binary suspensions. Bottom surface: at 0 particle interaction, mixed suspension is stable at low sphere and platelet concentration, but demixing occurs at high concentration; Surrounding surfaces: as particle interaction increases, particle aggregation would become more significant, and demixing would occur at lower particle concentrations; Top surface: particle interaction remarkable enough to decrease the stable region to the experimental case.

Here, we shall just qualitatively discuss the collaborative effects of both particle mixing and particle interaction on the colloidal stability of binary suspensions. A 3D schematic phase diagram is proposed in Fig. 4 to illustrate both the effects. It is a combination of phase diagrams of suspensions reported in the literature and our work here. The solid body in the 3D phase diagram represents the stable region. Outside surfaces of the 3D phase diagram are also presented for studying the effects of particle mixing or particle interaction. A limiting case for the binary suspension is a pure sphere suspension without depletion agent. The pure sphere suspension is stable at dilute concentrations while aggregation occurs above certain concentration, *m*_c_, which depends on the strength *U* of the interaction. A power law function was used indicating the boundary of *m*_c_ on *U*^32^. This phase transition is shown in the front surface of the phase diagram, which is consistent with the literature^32^. For binary suspensions, the stable region decreases with the increase in particle attraction, as shown in the 3D phase diagram. When the particle interaction is 0, the binary suspension is stable at low sphere and platelet concentrations; conversely, the suspension becomes unstable and demixing occurs at high concentration because of the depletion attraction of platelets and spheres. This situation is shown in the bottom surface of the phase diagram, which is consistent with the hard particle model with no attractive interactions^32^. However, with the particle interaction increase, particle aggregation would become significant. At high sphere or platelet concentrations, the stable region decreases remarkably because of both particle interaction and depletion, and a typical case is shown in the back surface. If particle interaction is high enough, the stable region decreases to the case as shown in the top surface, which is in accordance with the phase diagram as shown in Fig. 3 based on our experimental data. The findings suggest that both effects of particle mixing and particle interaction should be considered in the study of colloidal stability of binary suspensions.

## Methods

TiO_2_ spheres (Aladdin Industrial Co. Shanghai, China) were used as received, whereas TiO_2_ platelets were synthesized in our laboratory. During platelet synthesis, 10 mL titanate isopropoxide (Chengdu Ai Keda Chemical Technology Co., Ltd., China) was added into a 40 mL Teflon-lined autoclave; then, 2.4 mL of 12% hydrofluoric acid solution (Aladdin Industrial Co. Shanghai, China) was added drop-wise. The mixture was heated in a Teflon-lined stainless steel autoclave at 180 °C for 24 h. After cooling to room temperature, the white precipitate was washed with pure water for several times prior to drying at 60 °C overnight.

For suspension preparation, a certain amount of mixed sphere and platelet powders were added into deionized water. The mixture was stirred using a magnetic stirrer for 1 h to ensure that the powders are well-suspended in water. Then, the mixture was subjected to ultrasonic agitation for 1 h by using an ultrasonic cleaner, and the suspensions were prepared.

## Additional Information

**How to cite this article**: Mo, S. *et al.* Increasing entropy for colloidal stabilization. *Sci. Rep.* **6**, 36836; doi: 10.1038/srep36836 (2016).

**Publisher’s note:** Springer Nature remains neutral with regard to jurisdictional claims in published maps and institutional affiliations.
